# Tracking the Path of Migratory Pain: Unveiling Whipple Disease

**DOI:** 10.7759/cureus.91494

**Published:** 2025-09-02

**Authors:** Tiago Beirão, Luis Gomes, Catarina Silva, Catarina Rua, Mariana Patela, Margarida Caldas, David João, Flavio Costa, Patricia Pinto, Romana Vieira, Joana Aleixo-Santos, Ana Sofia Pinto, Luís Malheiro, Tiago Meirinhos, Taciana Videira

**Affiliations:** 1 Rheumatology Department, Local Health Unit Gaia and Espinho, Vila Nova de Gaia, PRT; 2 Gastroenterology Department, Portuguese Institute for Oncology of Lisbon, Lisbon, PRT; 3 Anatomic Pathology Department, Local Health Unit Gaia and Espinho, Vila Nova de Gaia, PRT; 4 Infectious Diseases Department, Local Health Unit Gaia and Espinho, Vila Nova De Gaia, PRT; 5 Rheumatology Department, Local Health Unit Gaia and Espinho, Vila Nova De Gaia, PRT

**Keywords:** endoscopy, gastrointestinal, mesenteric, spondylarthritis, whipple’s disease

## Abstract

Whipple’s disease (WD), a rare systemic infection caused by *Tropheryma whipplei*, often presents diagnostic challenges due to its nonspecific and varied clinical manifestations. We report the case of a woman initially diagnosed with undifferentiated connective tissue disease, who later developed severe malabsorptive symptoms, including steatorrhea and weight loss. Imaging revealed mesenteric lymphadenopathy, while histopathology and polymerase chain reaction (PCR) confirmed WD. Treatment included intravenous ceftriaxone followed by prolonged oral cotrimoxazole, leading to significant clinical improvement. However, persistent histological abnormalities warranted extended therapy. This case highlights the importance of considering infectious etiologies in atypical rheumatologic presentations, the role of molecular diagnostics in confirming WD, and the necessity of a multidisciplinary approach. Early recognition and appropriate management are critical to improving outcomes in this elusive disease.

## Introduction

Whipple's disease (WD) is a rare systemic condition primarily characterized by gastrointestinal manifestations such as diarrhea and malabsorption, often accompanied by polyarthralgia. With an estimated incidence of <1 per million annually, Whipple’s disease remains exceedingly rare and underdiagnosed. WD is more common in middle-aged Caucasian men, with a male-to-female ratio of approximately 8:1. However, the clinical presentation is highly variable, frequently resulting in delayed diagnosis [1]. The disease is caused by *Tropheryma whipplei*, a gram-positive actinomycete, first identified in 1907 and later characterized by molecular techniques in the 1990s, that predominantly affects the gastrointestinal tract, and less commonly extraintestinal sites, such as the central nervous system (dementia, ophthalmoplegia), ocular structures (uveitis), and cardiac tissue (endocarditis) [2]. The pathogenesis involves impaired bacterial clearance due to host immune dysfunction, particularly defective macrophage bactericidal activity, leading to widespread tissue infiltration. WD may occur in individuals with immune dysregulation, including associations with HLA-B27 and impaired macrophage function. Atypical presentations such as migratory arthralgia in the absence of classic gastrointestinal symptoms pose additional challenges for timely diagnosis, with a median time of six to seven years between musculoskeletal and gastrointestinal symptoms. Imaging modalities, including abdominal computed tomography (CT) and positron emission tomography-CT (PET/CT), may reveal findings such as mesenteric lymphadenitis. However, these features are nonspecific and can mimic various inflammatory, infectious, and neoplastic conditions [3]. Definitive diagnosis relies on histopathological analysis, with small bowel biopsies revealing characteristic features, such as villous distortion and macrophage infiltration. While Periodic acid-Schiff (PAS) staining remains a classical diagnostic tool, it lacks specificity, as other infections may yield false positives. Polymerase chain reaction (PCR) for *T. whipplei* provides higher sensitivity and specificity, enabling earlier diagnosis, particularly in atypical cases [4]. Treatment requires prolonged antibiotic therapy, yet relapse and persistent histological changes remain common challenges. Herein, we present a case of Whipple's disease initially misdiagnosed as an undifferentiated connective tissue disease, highlighting the importance of maintaining a broad differential diagnosis and the critical role of histopathological and molecular diagnostic techniques in uncovering this rare but significant condition.

## Case presentation

A woman in her 60s with a medical history of vesicular polyps presented to the rheumatology department with Raynaud phenomenon, xerostomia, and migratory inflammatory polyarthralgia of the wrists and knees. A physical examination revealed no evidence of arthritis. Immunological testing was positive for antinuclear antibodies (ANA) at a titer of 1:320 with a speckled pattern and negative for extractable nuclear antigen (ENA) antibodies. Further evaluation, including capillaroscopy and salivary gland biopsy, revealed no pathological abnormalities. Based on these findings, a diagnosis of undifferentiated connective tissue disease (UCTD) was established. The patient was started on hydroxychloroquine at a dose of 5 mg/kg/day, which led to the resolution of her complaints.

Four years later, the patient reported an eight-month history of chronic steatorrhea diarrhea, accompanied by significant weight loss exceeding 17 kg over a three-month period. The patient also presented with severe asthenia and anorexia. There were no associated symptoms, such as fever, chills, abdominal pain, or other systemic manifestations. Stool analyses, including tests for *Clostridium difficile* toxin, parasites, viruses, and fecal leukocytes, yielded negative results. Fat quantification was not performed.

**Figure 1:**
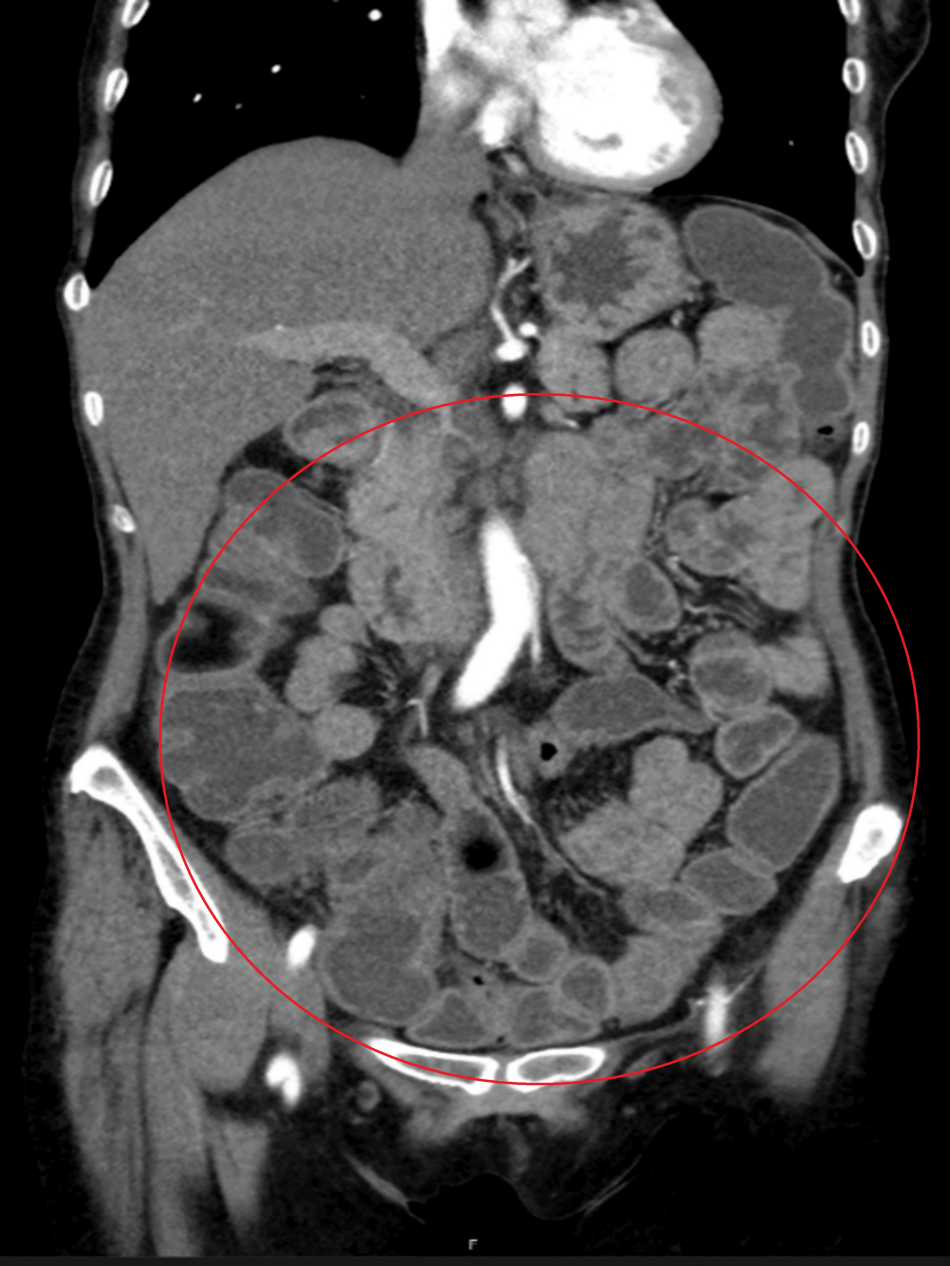
Abdominal CT scan: semi-distended small bowel loops with increased wall thickness, predominantly in the left hypochondrium and flank regions (red circle), at the beginning of the disease.

Chest CT scan revealed acute pulmonary thromboembolism with a Pulmonary Embolism Severity Index (PESI) score of 60 points (Class I, very low risk) [5]. Abdominal CT scan revealed semi-distended small bowel loops with increased wall thickness, predominantly in the left hypochondrium and flank regions (Figure 1). Prominent and enlarged mesenteric lymph nodes were observed, with the largest measuring 22 × 15 mm. Similar findings were identified around the celiac trunk, cephalopancreatic region, and hepatic hilum. 18F-Fluorodeoxyglucose positron emission tomography/computed tomography (18F-FDG PET/CT) demonstrated increased metabolic uptake in the small intestine, particularly in the left hemiabdomen, accompanied by adjacent mesenteric fat densification and nodular formations exhibiting mild hypermetabolism.

**Figure 2:**
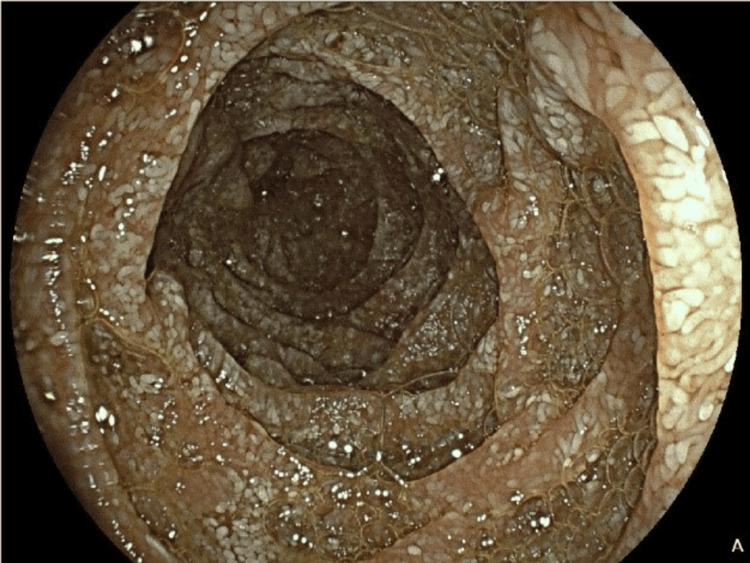
Esophagogastroduodenoscopy: duodenal lymphangiectasia, at the beginning of the disease.

**Figure 3:**
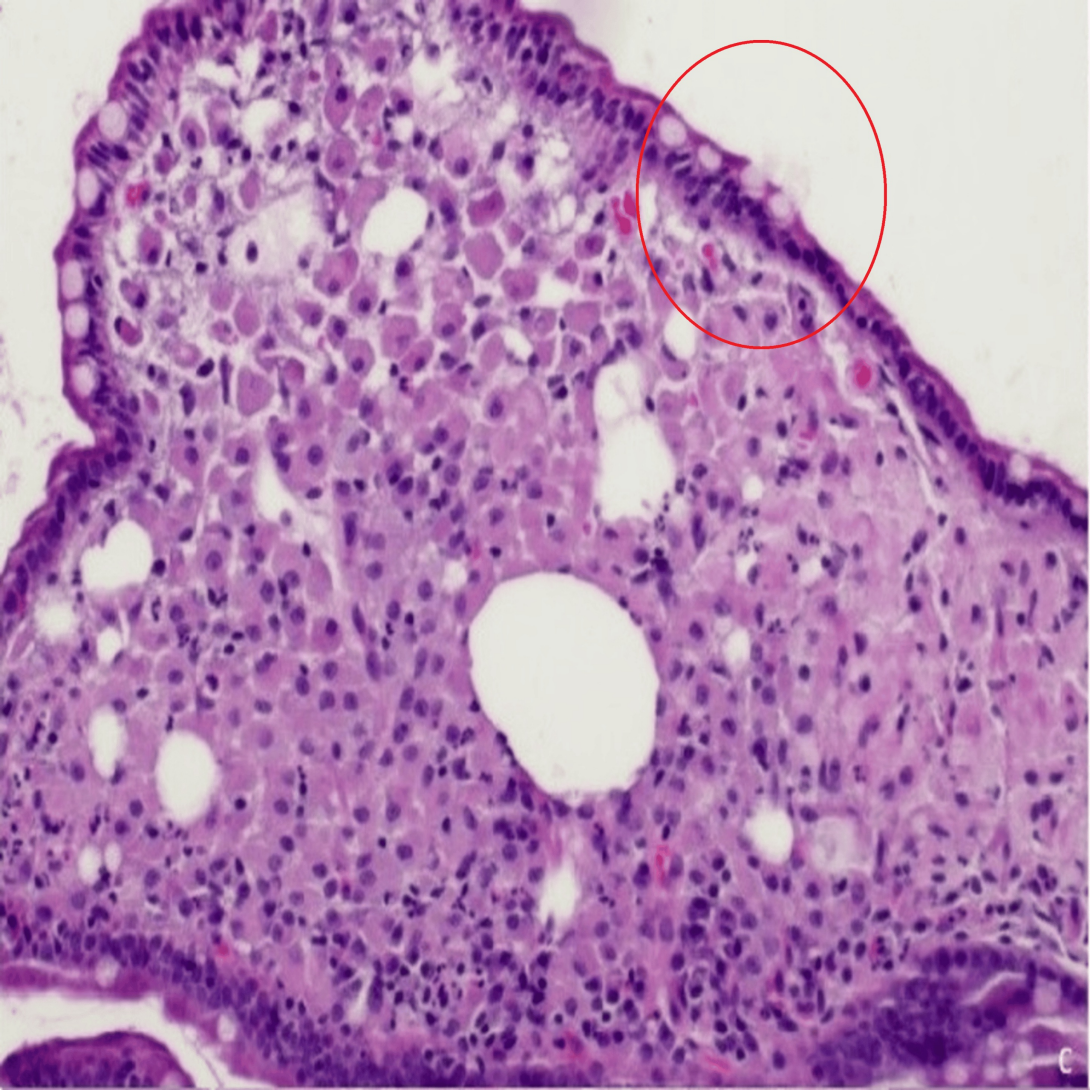
Duodenal biopsy: Periodic acid-Schiff (PAS) staining after diastase digestion revealed granular cytoplasmic positivity within macrophages (red circle).

Esophagogastroduodenoscopy (EGD) revealed no significant abnormalities in the esophagus or stomach; however, duodenal lymphangiectasia was identified in the D2 segment, and biopsies were subsequently obtained (Figure 2). Histological analysis of gastric biopsies demonstrated chronic gastritis without evidence of *Helicobacter pylori*. Four duodenal biopsies of D2 and D3 showed expansion of the lamina propria, villous architectural distortion, an inflammatory infiltrate predominantly composed of macrophages, and the presence of extracellular lipid droplets. Periodic acid-Schiff (PAS) staining after diastase digestion revealed granular cytoplasmic positivity within macrophages, while Ziehl-Neelsen staining did not detect acid-fast bacilli (Figure 3). Immunohistochemistry confirmed CD68 positivity in the macrophages. Polymerase chain reaction (PCR) testing of the biopsy sample for *Tropheryma whipplei* returned positive results. Brain magnetic resonance imaging (MRI) revealed no intracranial masses or vascular abnormalities, and a lumbar puncture was not performed as the patient exhibited no central or peripheral neurological symptoms. Transthoracic echocardiography did not raise suspicion for infective endocarditis.

**Figure 4:**
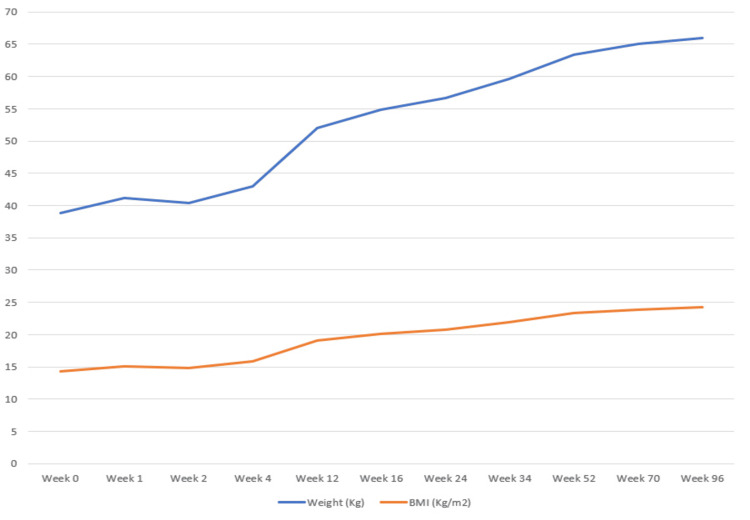
Evolution of weight throughout treatment. There was a stable weight in the first four weeks, with progressive increase until week 96.

The patient was transferred to the Infectious Diseases Department and commenced on intravenous ceftriaxone at a dose of 2 g daily for 28 days. Concurrent anticoagulation therapy with apixaban was initiated. At the onset of treatment, the patient exhibited severe malnutrition, characterized by significant weight loss (Figure 4), a body mass index (BMI) of 14.3 kg/m², hypoalbuminemia with peripheral edema, macrocytic anemia, hypocalcemia, hypomagnesemia, hypophosphatemia, and severe vitamin D deficiency. Other symptoms included peripheral arthralgia and moderate depression. Inflammatory biomarkers included an increased C-reactive protein and increased erythrocyte sedimentation rate. Clinical improvement was noted within the first week of therapy, including a reduction in the frequency of daily bowel movements, normalization of stool consistency, increased appetite, and marked alleviation of asthenia. Dietary supplementation was introduced to address malnutrition.

**Figure 5:**
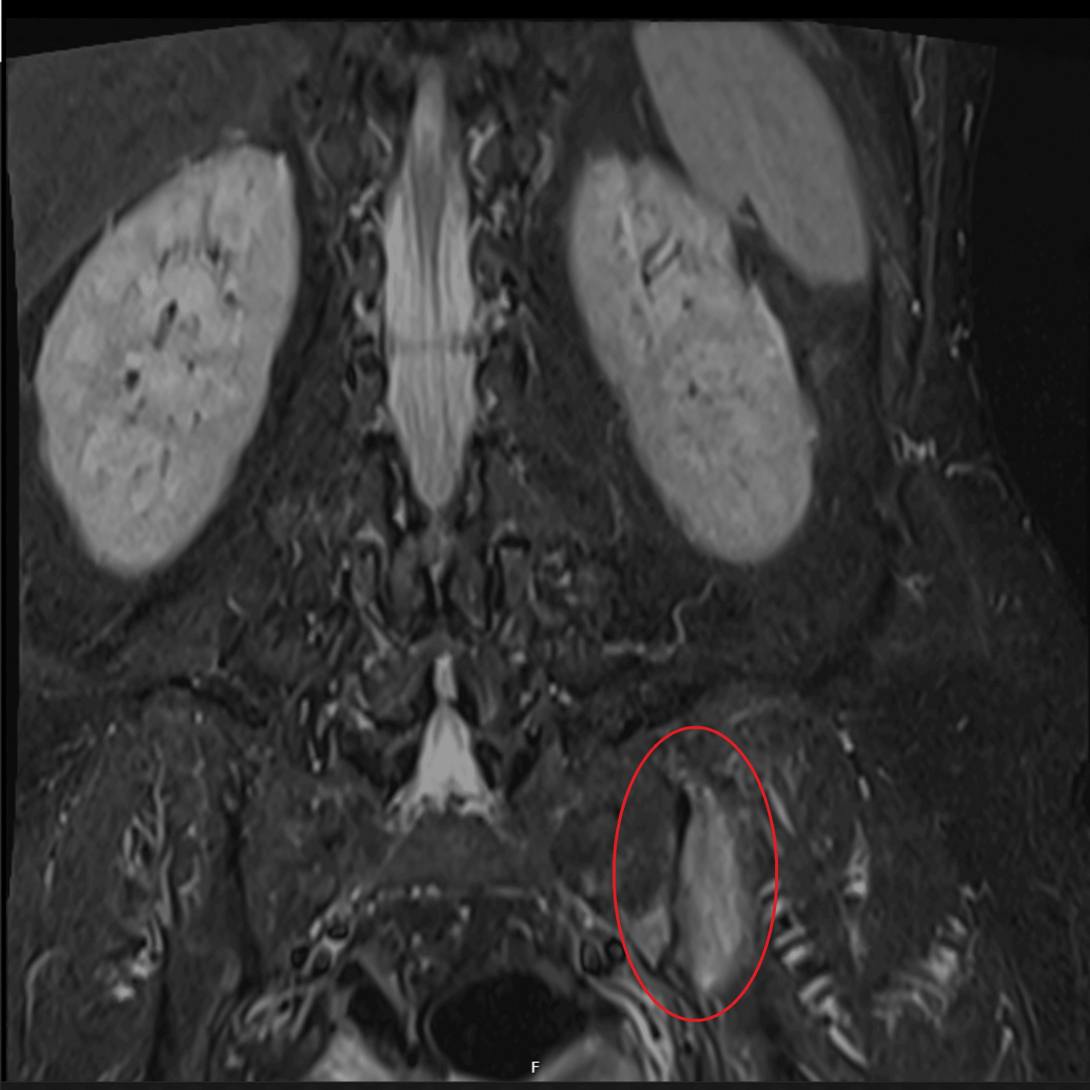
Lumbar MRI: asymmetric sacroiliitis (red circle).

Following the intravenous phase, the patient transitioned to oral trimethoprim-sulfamethoxazole at a dose of 960 mg every 12 hours. This regimen was well tolerated, with no adverse effects reported. Adherence to antimicrobial therapy was excellent, and BMI, along with clinical symptoms, improved significantly over subsequent months (Figure 4). Six months after initiating therapy, the patient developed persistent mechanical dorsal pain, and imaging confirmed spontaneous fractures of the dorsal vertebrae (D6 and D7), with diagnosis of malnutrition; glucocorticoid-free osteoporosis was made; and treatment with bisphosphonates was initiated. Furthermore, an MRI of the lumbar spine detected an asymmetric sacroiliitis (Figure 5), with no symptoms linked to it, and negative sacroiliac maneuvers. After one year of therapy, a repeat EGD revealed several areas of intestinal metaplasia in the gastric antrum, but no significant abnormalities in the duodenum. However, duodenal biopsies suggested persistent Whipple's disease, and PCR testing for *T. whipplei* remained positive. Based on these findings, the treatment regimen was extended for an additional year. It is worth noting that there are no standardized criteria for stopping therapy and that treatment duration is guided by symptom resolution, PCR clearance, and histology. Clinically, the patient showed sustained improvement and remained stable, continuing therapy with cotrimoxazole. Two years after the initiation of treatment, a follow-up EGD demonstrated no macroscopic abnormalities in the duodenum. Duodenal biopsies still demonstrated villous-type mucosa with preserved architecture and no significant inflammation, featuring scattered telangiectasias and aggregates of xanthomatous histiocytes along the villous axis. Spherical to slightly elongated particles, PAS-positive and variably Grocott-positive, were observed within these histiocytic aggregates. PCR testing for *T. whipplei* was negative, confirming eradication. Cotrimoxazole was suspended after 96 weeks of therapy.

## Discussion

Whipple’s disease (WD) presents a formidable diagnostic challenge due to its rarity and its propensity to mimic more common rheumatologic and systemic conditions. Caused by *Tropheryma whipplei*, a gram-positive actinobacterium with a trilamellar membrane, WD frequently manifests with nonspecific symptoms, most notably, polyarthralgia, that often appear early and overlap with features typical of autoimmune disorders [6]. In our patient, the initial diagnosis of undifferentiated connective tissue disease (UCTD) and the subsequent improvement with hydroxychloroquine underscore the inherent difficulties in recognizing WD. Notably, major malabsorptive symptoms did not emerge until four years after the initial musculoskeletal manifestations, emphasizing the need for ongoing clinical vigilance and periodic reassessment when the disease course deviates from that expected in UCTD [7].

A study conducted at a French reference center analyzed 142 patients with Whipple's disease, revealing that the interval between the onset of initial symptoms and the definitive diagnosis ranged from 0 to 30 years, with a mean of 6.4 years [8]. Notably, prior to receiving a conclusive diagnosis of Whipple's disease, 50% of patients had been treated for rheumatologic conditions, and in some cases, suspicion of lymphoma prompted histological examination. From a rheumatological perspective, new gastrointestinal symptoms could be attributed to an adverse effect of long-term hydroxychloroquine use, a new comorbidity, or a manifestation of systemic autoimmune disease such as Crohn’s disease or ulcerative colitis [9]. However, significant weight loss and chronic diarrhea are more consistent with a malabsorptive disorder, and findings such as small-bowel wall thickening and prominent mesenteric lymph nodes are also unusual in inflammatory colitides [10,11]. Although autoimmune conditions with gastrointestinal involvement (e.g., vasculitis or connective tissue disease-related enteropathy) might be considered, the final diagnosis in this case emphasizes the importance of maintaining an open differential that includes infectious etiologies, particularly when systemic and malabsorptive symptoms are present. Furthermore, numerous reports have documented a complicated course of WD following medical immunosuppression, especially after treatment with tumor necrosis factor (TNF) inhibitors, which are also commonly employed in managing rheumatologic disorders [12].

The diagnostic breakthrough was achieved through histological examination of duodenal biopsies, which revealed villous architectural distortion, a macrophage-rich inflammatory infiltrate, and PAS-positive granules, findings highly suggestive of WD [13]. Confirmation by qualitative PCR testing for *T. whipplei* further underscores the critical role of molecular diagnostics in establishing the diagnosis. It is worth mentioning that mesenteric lymphadenopathy and PET-avid bowel loops are atypical for classic IBD and may favor WD or lymphoma.

Given the rarity of the disease, evidence for the best treatment choices is limited to a few clinical trials and observational studies, with no randomized controlled trials. It has long been proposed that for classic WD without CNS involvement, therapeutic intervention should start with intravenous ceftriaxone [14]. The patient subsequently transitioned to long-term oral trimethoprim-sulfamethoxazole (SMX/TMP), a regimen that has been proven effective for maintaining remission and preventing relapse [15]. However, observational studies have found conflicting results with SMX/TMP, with some series revealing high on-treatment failures and relapses and other combinations such as doxycycline with hydroxychloroquine for 12 months followed by lifelong suppression with doxycycline have been suggested [16]. In-vitro studies raise doubts about the effectiveness of SMX/TMP administration, given *T. whipplei* lack of susceptibility to trimethoprim. Furthermore, the potential for developing resistance to sulfamethoxazole presents additional risks, making the use of this treatment approach questionable [17].

Treatment duration and the decision to discontinue antimicrobial therapy remain challenging in the management of Whipple’s disease. Despite initial clinical improvement, follow-up endoscopy at one year revealed persistent duodenal abnormalities, and PCR testing for *Tropheryma whipplei* remained positive. These findings underscore the difficulties in achieving complete remission in WD and highlight the need for extended therapy, ongoing surveillance, and repeated diagnostic assessments when clinical or histological remission is incomplete [2]. In instances of relapse or when initial antibiotic regimens prove insufficient, a combination of doxycycline with hydroxychloroquine may be a viable alternative. In our case, follow-up endoscopy after 24 months of treatment demonstrated macroscopic resolution; however, duodenal biopsies continued to show histopathologic changes suggestive of WD despite a negative PCR for *T. whipplei*. It is important to note that the persistence of PAS-positive macrophages may continue for years following successful eradication of the bacterium and does not necessarily indicate insufficient treatment [18]. Long-term monitoring (clinical + molecular) is recommended because relapses can occur even >10 years later.

Lastly, the management of Whipple’s disease mandates a multidisciplinary approach involving infectious disease specialists, gastroenterologists, rheumatologists, nutritionists, and endocrinologists. In our case, comprehensive nutritional assessment and targeted dietary interventions were essential to address severe malnourishment and multiple micronutrient deficiencies, which contributed to secondary osteoporosis and subsequent vertebral fractures. Additionally, vigilant monitoring for immune reconstitution inflammatory syndrome (IRIS) is critical, as the initiation of effective antimicrobial therapy may precipitate paradoxical inflammatory responses [19]. Close surveillance of bone health is equally important to promptly identify and manage osteoporotic complications. This integrated strategy, addressing both the primary infection and its systemic sequelae, highlights the necessity of a coordinated, multidisciplinary approach to optimize patient outcomes in complex cases of Whipple’s disease.

## Conclusions

Rheumatologists should maintain a high index of suspicion for atypical clinical presentations that deviate from classic autoimmune disease patterns. In such cases, it is essential to broaden the differential diagnosis to include infectious causes, particularly rare pathogens like *Tropheryma whipplei*. This is especially important because Whipple disease can mimic various rheumatologic conditions, potentially leading to misdiagnosis and delayed treatment. A routine reconsideration of WD in patients with systemic features and unexplained GI involvement is recommended, especially those on immunosuppression or with ANA positivity but incomplete rheumatologic workup. The use of a multidisciplinary diagnostic approach that combines detailed histopathological examination with advanced molecular techniques, such as polymerase chain reaction (PCR) testing for *T. whipplei*, is fundamental to establishing an accurate and timely diagnosis. Maintaining a high index of suspicion for Whipple’s disease in atypical or refractory cases is critical to preventing misdiagnosis, avoiding unnecessary immunosuppression, and ensuring timely, life-saving treatment, especially in atypical clinical presentations.
